# Evaluation of the *In Vitro* and *In Vivo* Antioxidant Potentials of *Aframomum melegueta* Methanolic Seed Extract

**DOI:** 10.1155/2014/159343

**Published:** 2014-05-15

**Authors:** Samuel Okwudili Onoja, Yusuf Ndukaku Omeh, Maxwell Ikechukwu Ezeja, Martins Ndubuisi Chukwu

**Affiliations:** ^1^Department of Veterinary Physiology, Pharmacology, Biochemistry and Animal Health and Production, College of Veterinary Medicine, Micheal Okpara University of Agriculture, PMB 7267, Umudike, Nigeria; ^2^Department of Biochemistry, College of Natural Sciences, Micheal Okpara University of Agriculture, PMB 7267, Umudike, Nigeria

## Abstract

*Aframomum melegueta* Schum (Zingiberaceae) is a perennial herb widely cultivated for its valuable seeds in the tropical region of Africa. The present study evaluated the antioxidant effects of methanolic seed extract of *A. melegueta*. The antioxidant effects were evaluated using *in vitro*, 2, 2-diphenylpicrylhydrazine photometric assay and *in vivo* serum catalase, superoxide dismutase and thiobarbituric acid reactive substance assay method. The extract (25–400 **μ**g/mL concentration) produced concentration dependent increase in antioxidant activity in 2, 2-diphenylpicrylhydrazine photometric assay. The extract (400 mg/kg) showed a significant (*P* < 0.05) increase in serum catalase and superoxide dismutase activity when compared with the control group. The extract (400 mg/kg) showed a significant (*P* < 0.05) decrease in the serum level of thiobarbituric acid reactive substance when compared with the control group. These findings suggest that the seed of *A. melegueta* has potent antioxidant activity which may be responsible for some of its reported pharmacological activities and can be used as antioxidant supplement.

## 1. Introduction

Antioxidants act as a defence mechanism that protect against deleterious effects of oxidative reaction produced by reactive oxygen species (ROS) in a biological system [1]. Reactive oxygen species not only are produced naturally in cell following stress or respiration but also have been reported to be produced by radiation, bacterial and viral toxin, smoking, alcohol, and psychological or emotional stress. Overproduction of ROS and/or inadequate antioxidants has been implicated in the pathogenesis and complications of some disease conditions like diabetes, Alzheimer's disease, cancer, atherosclerosis, arthritis, neurodegenerative disease, and aging process [2, 3]. Antioxidants have been reported to prevent oxidative damage caused by ROS by reacting with free radicals, chelating, and catalytic metals and also by acting as oxygen scavengers [4, 5]. The antioxidants in biological system can be either enzymatic or nonenzymatic. The enzymatic antioxidants include catalase, superoxide dismutase, and glutathione which catalyse neutralization of many types of free radicals [6], while the nonenzymatic antioxidants include Vitamin C, selenium, vitamin E, carotenoids, and polyphenols. There is growing evidence that antioxidants play a pivotal role in the prevention of heart disease, cancer, DNA degeneration, pulmonary disease, and neurological disorder [7]. Recently, there has been an upsurge of interest in the therapeutic potential of plants as antioxidants in reducing oxidative tissue injuries [3]. Plants, herbs, and spice, rich in phenolic compounds like flavonoids, have been demonstrated to have anti-inflammatory, antiallergenic, antiviral, antiaging, and anticarcinogenic activities which can be attributed to their antioxidant properties [7, 8].

*Aframomum melegueta *Schum (Zingiberaceae) also known as Guinea pepper, grains of paradise, or alligator pepper (indigenous names include Atare in Yoruba, Ose-oji in Igbo, and Citta in Hausa) is a perennial herb widely cultivated for its valuable seeds in the tropical region of Africa [9, 10]. It grows up to 1.5 m in height, with purple flower that develop into long pod containing small, reddish brown aromatic and pungent seed. In Nigeria and some other parts of West Africa, the seeds are used as a spicy and have a wide range of folkloric uses in traditional medicine. They are used as a remedy for treating stomach ache, diarrhoea, and snakebite [9, 10]. Previous studies have established the antiulcer, antimicrobial, anti-inflammatory, and sexual performance enhancing effects of the seed extract [9–12]. The seeds are very rich in the nonvolatile pungent compounds gingerol, shogaols, paradol, and related compounds [11]. The present study aimed at establishing the* in vitro* and* in vivo* antioxidant potentials of the methanolic seed extract of* Aframomum melegueta.*

## 2. Materials and Methods

### 2.1. Plant

The freshly harvested fruit of* Aframomum melegueta* (Alligator Pepper) were bought from Ndoro market, Oboro in Ikwuano LGA of Abia State in the month of July 2013, and were authenticated by Dr. I. C. Okwulehie of the Department of Plant Science and Biotechnology, Michael Okpara University of Agriculture, Umudike, and the voucher specimen catalogued MOUAU/CVM/VPP/2013/03 was kept for reference purpose in the departmental herbarium.

### 2.2. Preparation of the Extract

Dried and pulverized seeds of* Aframomum melegueta* were extracted by cold maceration method for 48 hours at room temperature using absolute methanol in a Winchester bottle. The* Aframomum melegueta *extract (AME) was filtered with Whatman No. 1 filter paper. The filtrate was concentrated* in vacuo* using vacuum rotary evaporator at 40°C and was later concentrated to dryness in a hot-air oven at 40°C. The extract was stored in a refrigerator at 4°C throughout the duration of this study.

### 2.3. Determination of the Yield of the AME

An empty clean and dry beaker was weighed and later the extract was poured into it. The beaker was weighed after the extract has been concentrated to constant weight. The weight of the extract was calculated as follows:

(1) The  percentage  yield  of  extract(%)w/w=weight  of  beaker  and  extract−wieght  of  empty  beaker  weight  of  plant  material ×1001.

### 2.4. Animals

Twenty male Wistar albino rats weighing between 120 and 170 g were obtained from Department of Zoology, University of Nigeria, Nsukka, and kept in the Animal House of the Biochemistry Department. The animals were allowed access to feed and water* ad libitum *and were allowed two weeks to acclimatize before the commencement of the experiment. The animals were kept in well-ventilated aluminium cages at room temperature and under natural light/darkness cycles. They were maintained in accordance with the recommendation of the* Guide for the care and use of laboratory animals* [13]. The experiment was approved by the University Animal Ethics Committee with reference MOUAU/CVM/EAEC/2013/201.

### 2.5. Phytochemical Spot Test

The AME was tested for the presence of alkaloids, flavonoids, tannins, glycosides, saponins, terpenes/sterols, carbohydrates, and starch using the standard procedures as described by Trease and Evans [14].

### 2.6. Determination of the* In Vitro* Antioxidant Activities of AME Using 2, 2-Diphenyl-1-picrylhydrazyl (DPPH) Photometric Assay

The free radical scavenging activity of the extract was analyzed by DPPH assay using spectrophotometer [15]. Each of the test extracts (2 mL) at different concentrations (25, 50, 100, 200, and 400 *μ*g/mL) was mixed with 0.5 mM DPPH (in 1 mL of methanol) in a cuvette. The absorbance at 517 nm was taken after 30 minutes of incubation in the dark at room temperature. The concentrations were prepared in triplicates and the percentage antioxidant activity calculated as follows:

(2) %  antioxidant  activity  (AA)=100−[{(absorbance  of  sample−absorbance  of  blank)     ×100}×(absorbance  of  control)−1].

One milliliter of methanol plus 2.0 mL of the extract was used as the blank, while 1.0 mL of the 0.5 mM DPPH solution plus 2.0 mL of methanol was used as the negative control. Ascorbic acid (vitamin C) was used as reference standard [16].

### 2.7. Determination of the* In Vivo* Antioxidant Effect of AME

Twenty male albino Wistar rats were randomly divided into four groups of five animals each. Group 1 served as the control and received 0.4 mL of distilled water. Group 2 received 100 mg/kg of the AME. Group 3 received 200 m/kg of the AME, and group 4 received 400 mg/kg of the AME. The animals were dosed daily for 21 days and were observed daily for changes and other signs of toxicity and death throughout the period of study. Twenty-four hours after the last treatment, blood obtained through direct cardiac puncture was used to assay for* in vivo* antioxidant activity of AME.

### 2.8. Analytical Methods

#### 2.8.1. Serum Preparation

The blood used for serum preparation was collected via direct heart puncture with 21 G needle attached to 5 mL syringe, following mild chloroform anaesthesia of the rats. The serum was prepared using standard method as described by Yesufu et al. [17]. Briefly, the method used is as follows. Blood was allowed to clot for 30 minutes and then centrifuged at 2500 rpm for 15 minutes and serum was harvested.

#### 2.8.2. Determination of the Lipid Peroxidation (LPO) in Serum

The level of thiobarbituric acid reactive substance (TBARS) and malondialdehyde (MDA) production was measured in serum by the modified method as described by Draper and Hadley [18]. The serum (50 *μ*L) was deproteinized by adding 1 mL of 14% trichloroacetic acid and 1 mL of 0.6% thiobarbituric acid. The mixture was heated in a water bath for 30 min to complete the reaction and then cooled on ice for 5 min. After centrifugation at 2000 g for 10 min, the absorbance of the colored product (TBARS) was measured at 535 nm with a UV spectrophotometer. The concentration of TBARS was calculated using the molar extinction coefficient of malondialdehyde (1.56 × 10^5^ mol/L/cm) using the formula, A = ΣCL, where A = absorbance, Σ = molar coefficient, C = concentration, and L = path length. The results were expressed in nmol/mg of protein.

#### 2.8.3. Estimation of Superoxide Dismutase (SOD)

Superoxide dismutase activity was assayed according to the method of Sun et al. [19]. In this method, xanthine-xanthine oxidase system was used to generate a superoxide flux, and nitroblue tetrazolium (NBT) was used as an indicator of superoxide production. SOD activity was then measured by the degree of inhibition of the reaction unit of enzyme providing 50% inhibition of NBT reduction. Results are expressed as U/mL.

#### 2.8.4. Estimation of Catalase Activity

The catalase activity in serum was determined using the modified method as described by Atawodi [20]. Briefly, the method is as follows: serum (10 *μ*L) was added to test tube containing 2.80 mL of 50 mM potassium phosphate buffer (pH 7.0). The reaction was initiated by adding 0.1 mL of fresh 30 mM hydrogen peroxide and the decomposition rate of hydrogen peroxide was measured at 240 nm for 5 min on a spectrophotometer. A molar extinction coefficient of 0.041 mM^−1 ^cm^−1^ was used to calculate catalase activity.

#### 2.8.5. Determination of Protein

The total protein content of the serum was assayed using commercially available total protein kit (Randox Laboratories, UK), employing direct Biuret method.

### 2.9. Statistical Analysis

Data obtained were analyzed using one-way analysis of variance (ANOVA) and the variant mean was separated by least significant difference (LSD) of the different groups. Significance was accepted at the level of *P* < 0.05.

## 3. Result

### 3.1. Phytochemical Spot Test

The phytochemical spot test showed that the extract contained saponins, tannins, terpenes/sterols, glycosides, alkaloids, and flavonoids.

### 3.2. The* In Vitro* Antioxidant Activity of AME Using DPPH Photometric Assay

**Figure 1:**
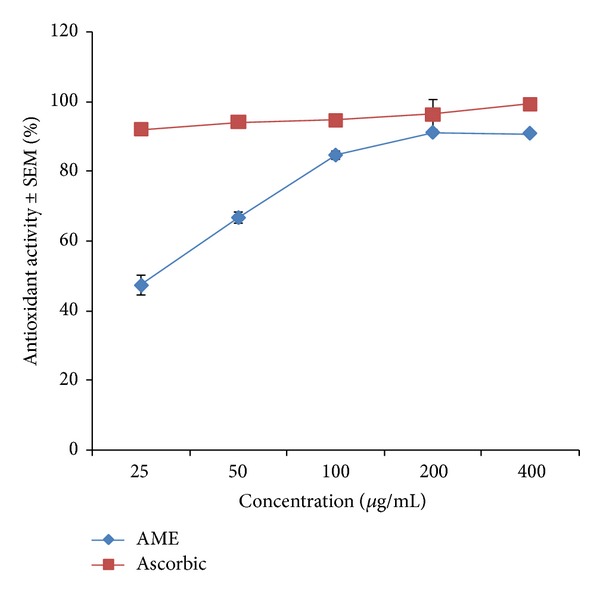
The* in vitro* antioxidant activity of AME using DPPH photometric assay.

The result of the* in vitro* antioxidant activity of AME is present in Figure 1. The extract produced concentration dependent increase in percentage antioxidant activity in DPPH spectrophotometric assay. The optimum activity was observed at 400 *μ*g/mL concentration of the extract.

### 3.3. The Effects of AME on the Body Weight of the Treated Rats

The result of the effects of AME on the body weight of the treated rats is presented in Table 1. The extract (all doses) did not produce any significant (*P* > 0.05) difference in body weight gain in treated rats when compared to the negative control.

### 3.4. The* In Vivo* Antioxidant Effects of AME in Rats

The results of the* in vivo* antioxidant effect of AME on rats are presented in Table 2. The extract produced a dose dependent decrease in the MDA levels in the serum. The serum MDA level of the group treated with 400 mg/kg of AME was significantly (*P* < 0.05) lower when compared to other treatment groups and the negative control group. The extract also produced a dose dependent increase in the serum level of catalase activity. The serum catalase activity of the group treated with 400 mg/kg of AME was significantly (*P* < 0.05) higher when compared to other treatment groups and the negative control group. Furthermore, the extract produced dose dependent increase in the serum level of superoxide dismutase activity. The serum superoxide dismutase activity of the groups treated with AME were significantly (*P* < 0.05) higher when compared with the negative control group.

## 4. Discussion

Antioxidants (free radical scavengers) are chemicals that interact with and neutralize free radicals, thus preventing them from causing cellular damage in the biological system [21]. The body makes some of the antioxidants it uses to neutralize free radicals. These antioxidants are called endogenous antioxidants. However, the body also relies on external (exogenous) sources, primarily the diet, to obtain the rest of the antioxidants it needs [22]. These exogenous antioxidants are commonly called dietary antioxidants. Fruits, vegetables, and grains are rich sources of dietary antioxidants [23].

The* in vitro* antioxidant potential of AME was assayed using DPPH photometric assay, while the* in vivo* antioxidant potential was evaluated using serum superoxide dismutase, catalase activity, and malondialdehyde level assay. The choice of the doses used in this study was based on previous work done by Umukoro and Ashorobi [9].

The* in vitro* antioxidant assay of AME revealed that it has a potent antioxidant activity comparable to vitamin C which was used as a reference standard. 2, 2-diphenyl-1-picrylhydrazyl (DPPH) is a dark-coloured crystalline powder composed of stable free radical molecules. In laboratory, it is used to monitor chemical reactions involving radicals, most notably antioxidant assay [24, 25]. The antioxidant compounds neutralize the free radical character of DPPH by transferring either electrons or hydrogen atoms to DPPH [26], thereby changing the colour from purple to the yellow coloured stable diamagnetic molecule diphenylpicrylhydrazine. The degree of discoloration indicates the scavenging potential of the extract or antioxidant in terms of hydrogen donating ability [27].

The* in vivo *antioxidant assay showed that the extract increased the activity of serum superoxide dismutase (SOD) and catalase and decreased the serum level of TBARS. Catalase is a ubiquitous enzyme that catalyzes the decomposition of hydrogen peroxide, a reactive oxygen species, which is a toxic product of both normal aerobic metabolism and pathogenic ROS production [28, 29]. The SOD catalyzes the dismutation of superoxide to hydrogen peroxide and oxygen, thereby reducing the likelihood of superoxide anion reacting with nitric oxide to form reactive peroxynitrite [29]. The increased serum activities of catalase and SOD as observed in this study suggest that the extract has an* in vivo* antioxidant activity and is capable of ameliorating the effect of ROS in biologic system [30, 31].

Also, ROS react with all biological substance; however, the most susceptible ones are polyunsaturated fatty acids. Reactions with these cell membrane constituents lead to lipid peroxidation (LPO) [31]. Increased LPO impairs membrane function by decreasing membrane fluidity and changing the activity of membrane-bound enzymes and receptor [32]. Thiobarbituric acid reactive substance (TBARS) levels were measured as a marker of LPO and malondialdehyde (MDA) production. Malondialdehyde is an endogenous genotoxic product of enzymatic and ROS-induced LPO whose adducts are known to exist in DNA isolated from healthy human being [33]. In our study, the level of TBARS in the extract treated groups decreased in a dose dependent manner when compared to control. This decrease in the TBARS levels may indicate increase in the activities of glutathione peroxidase and hence inactivation of LPO reactions [34].

Some of the phytochemical constituents of the extract may be responsible for the antioxidant activity as demonstrated in our study. Flavonoids or bioflavonoids are a ubiquitous group of polyphenolic substances which are present in most plants, concentrated in seeds, fruit skin or peel, bark, and flowers [35]. Numerous studies have shown that flavonoids possess potent antioxidant activities capable of scavenging hydroxyl radicals, superoxide anions, and lipid peroxy radicals [36]. Shahidi et al. [4] attributed the pharmacological activities (anti-inflammatory, antiviral, antibacterial, antiulcer, antiosteoporotic, antiallergic, and antihepatotoxic actions) of flavonoids to their potent antioxidant activity.

In conclusion, the demonstrated antioxidant and antilipid peroxidation effects of the extract of* A. melegueta* seed may be the rationale behind some of its folkloric uses and also may be responsible for some of its pharmacological effects.

## Figures and Tables

**Table 1:** The effects of AME on the body weight of the treated rats.

Group	Mean body weight in gram ± SEM (percentage increase in weight compared to day 0)			
	Day 0	Day 7	Day 14	Day 21
Distilled water 10 mL/kg	147.14 ± 4.09	163.96 ± 1.41 (11.74)	169.12 ± 2.05 (15.22)	174.02 ± 2.80 (18.49)
AME 100 mg/kg	134.24 ± 11.37	150.13 ± 12.50 (12.14)	160.94 ± 12.16 (20.32)	169.54 ± 13.31 (26.63)
AME 200 mg/kg	138.38 ± 6.61	152.52 ± 5.54 (10.57)	153.94 ± 5.07 (11.71)	156.18 ± 4.66 (13.54)
AME 400 mg/kg	149.38 ± 10.14	161.15 ± 10.71 (7.92)	165.58 ± 9.14 (11.22)	170.55 ± 7.36 (14.96)

No statistical difference (*P* > 0.05) compared with control group.

**Table 2:** The *in vivo* antioxidant effects of AME in rats (mean ± SEM).

Group	Catalase (*μ*mol/mg protein)	MDA (nmol/mg protein)	SOD (unit/mL)
Distilled water 10 mL/kg	16.80 ± 10.84	0.104 ± 0.01	11.00 ± 0.58
AME 100 mg/kg	35.29 ± 11.70	0.103 ± 0.05	14.00 ± 0.58*
AME 200 mg/kg	47.51 ± 20.90	0.086 ± 0.03	18.00 ± 0.58*
AME 400 mg/kg	128.72 ± 31.27*	0.016 ± 0.01*	20.33 ± 0.88*

*P < 0.05 are statistically significant when compared to distilled water treated group.
